# Immune Evasion Mechanism Mediated by ITPRIPL1 and Its Prognostic Implications in Glioma

**DOI:** 10.1002/brb3.70762

**Published:** 2025-08-12

**Authors:** Zou Xiaoyun, Ye Wenhao, Wu Huan, Yang Yuanyuan, Liu Changqing, Wen Hebao, Ma Caiyun

**Affiliations:** ^1^ Anhui Engineering Research Center for Neural Regeneration Technology and Medical New Materials Bengbu Medical University Bengbu China; ^2^ School of Life Science Bengbu Medical University Bengbu China; ^3^ Department of Physical Education and Arts Bengbu Medical University Bengbu China

**Keywords:** glioma, immune infiltration, ITPRIPL1, machine learning, prognostic biomarker

## Abstract

**Background:**

Glioma represent one of the most prevalent and lethal malignancies within the central nervous system. Recent studies have identified ITPRIPL1, a newly reported CD3ε‐inhibitory ligand, as a suppressor of T cell activation, thereby facilitating tumor immune evasion and offering a novel avenue for immunotherapeutic intervention in glioma.

**Methods:**

A comprehensive analysis was performed using datasets from The Cancer Genome Atlas (TCGA), the Chinese Glioma Genome Atlas (CGGA), and Gene Expression Omnibus (GEO). This included evaluating ITPRIPL1 expression levels in glioma, its association with clinicopathological features, prognostic significance, immune landscape, targeted drug sensitivity, and underlying biological functions. Ninety‐eight machine learning algorithm combinations were screened to identify the optimal predictive model. A nomogram was subsequently constructed and validated to assess the integrated prognostic impact of ITPRIPL1 expression on glioma patients.

**Results:**

Elevated ITPRIPL1 expression was positively correlated with higher tumor grade and poorer clinical outcomes. Immune infiltration profiling revealed that ITPRIPL1 expression was negatively associated with effector memory CD4⁺ T cells and type 17 T helper cells (Th17), but positively correlated with M2‐polarized macrophages and several immune checkpoint molecules. Moreover, drug sensitivity analyses and molecular docking studies highlighted a potential therapeutic relationship between ITPRIPL1 and antitumor agents such as AZD8055. The SuperPC model emerged as the most robust predictor and was utilized to develop a prognostic nomogram capable of reliably forecasting survival in glioma patients.

**Conclusions:**

This study reveals that ITPRIPL1 plays a dual role in glioma: It suppresses T cell–mediated immune responses, contributing to an immunosuppressive microenvironment, and interferes with the efficacy of antitumor drugs, thereby promoting tumor progression and ultimately leading to poor patient prognosis.

## Graphical abstract

A comprehensive analysis was conducted using the TCGA, CGGA and GEO databases, covering the expression level of ITPRIPL1 in glioma, clinical characteristics, prognostic value, immunological value, targeted drug screening, and biological functions. A predictive model was constructed through machine learning to assess its impact on the survival of glioma patients.

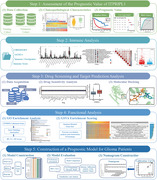

## 1 Introduction

Glioma is the most common and highly fatal primary brain tumors in adults, accounting for approximately 81% of all malignant brain tumors (Bray et al. 2024; Ostrom et al. 2023). Among them, glioblastoma (GBM), classified as WHO grade IV, represents the most aggressive subtype, constituting nearly 50% of all glioma cases. GBM is characterized by pronounced invasiveness and extremely poor prognosis (Sung et al. 2021; Yasinjan et al. 2023). Current standard‐of‐care regimens include neuronavigation‐guided surgical resection followed by radiotherapy and temozolomide‐based chemotherapy. However, the majority of patients eventually experience recurrence, malignant progression, and resistance to chemotherapeutic agents (Bray et al. 2024; X. Xu et al. 2021). Statistically, patients with grade III glioma exhibit a median survival of 3–5 years following treatment, whereas GBM patients, despite aggressive therapeutic intervention, have a median survival of only around 15 months (Fathi Kazerooni et al. 2021; Louis et al. 2021). These data underscore the urgent need for more effective treatment strategies for glioma.

In recent years, the advent of immunotherapy has brought renewed hope to clinical oncology, demonstrating potential in prolonging overall survival (OS) in patients with advanced glioma in clinical trials (Daubon et al. 2020; S. Xu et al. 2020). Immune checkpoint therapy (ICT), a cornerstone of immunotherapeutic strategies, enhances antitumor immunity by blocking inhibitory signals that suppress T cell activation (Sharma et al. 2023; Ye et al. 2025) However, the efficacy of immunotherapy in glioma is often hindered by immune evasion within the tumor microenvironment (TME), particularly due to T cell dysfunction (Koikawa et al. 2021; Oliveira and Wu 2023). CD3ε, a core component of the TCR‐CD3 complex, serves as a critical antibody target regulating T cell function (Sušac et al. 2022; Wunderlich et al. 2014). In 2024, Deng et al. reported in cell that ITPRIPL1 functions as an endogenous inhibitory ligand of CD3ε. By binding to CD3ε on the surface of T cells, ITPRIPL1 suppresses T cell activation and promotes tumor immune evasion. Notably, ITPRIPL1‐targeting antibodies exhibit potential to overcome resistance to immunotherapy, offering a novel therapeutic avenue for patients unresponsive to PD‐1/PD‐L1 inhibitors (Deng et al. 2024). These findings highlight the clinical significance of ITPRIPL1 in glioma immunotherapy. Therefore, elucidating the immunomodulatory role of ITPRIPL1 in glioma may offer valuable insights for diagnosis and therapeutic intervention.

**FIGURE 1:**
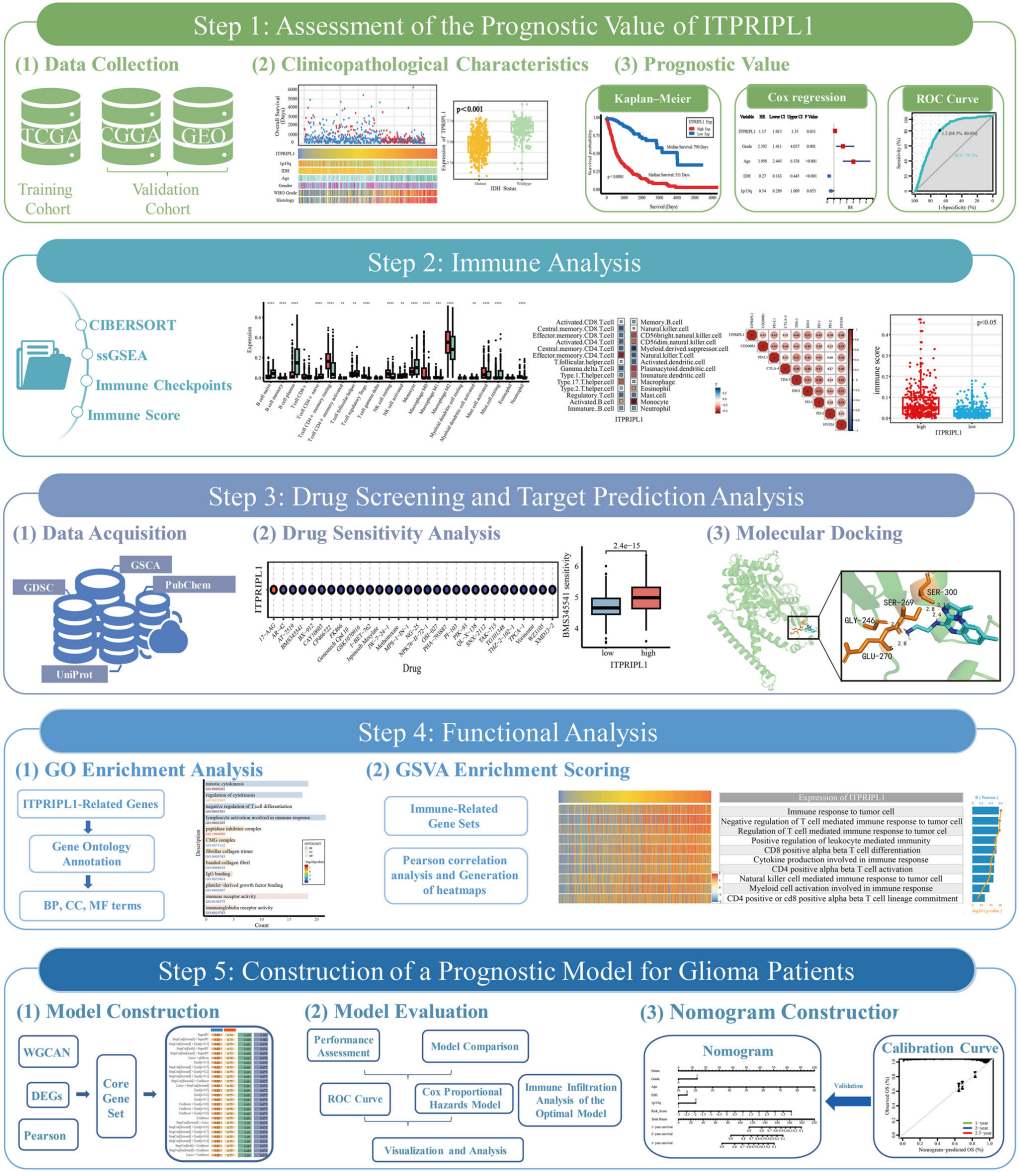
Overall workflow of the study.

This study systematically investigated the role of ITPRIPL1 in glioma using datasets from TCGA, CGGA, and GEO (Figure 1). First, the expression profile and prognostic significance of ITPRIPL1 across various clinical subgroups were evaluated. Subsequently, its association with the tumor immune microenvironment was examined using multiple immune infiltration algorithms. Drug sensitivity analysis and molecular docking were conducted based on data from Gene Set Cancer Analysis (GSCA), Genomics of Drug Sensitivity in Cancer (GDSC), UniProt, and PubChem databases to explore the therapeutic potential of ITPRIPL1 as a drug target. In addition, ITPRIPL1‐associated genes were identified and subjected to functional enrichment analysis. Finally, the optimal predictive model was constructed using machine learning algorithms, and a prognostic nomogram was developed to effectively estimate survival outcomes in glioma patients.

## 2 Materials and Methods

### 2.1 Data Collection and Preprocessing

RNA‐seq transcriptomic data and corresponding clinical information of glioma patients used in this study (Table 1) were obtained from the TCGA (https://www.cancer.gov/ccg/research/genome‐sequencing/tcga), CGGA (http://www.cgga.org.cn/), and GEO (https://www.ncbi.nlm.nih.gov/) databases. Multiple datasets were retrieved from CGGA, including CGGA.20 (non‐glioma as control, *n* = 20), CGGA.325 (*n* = 325), CGGA.693 (*n* = 693), and CGGA.array (*n* = 301). The validation cohort CGGA.1018 (*n* = 1018) was generated by merging CGGA.325 and CGGA.693 using principal component analysis (PCA), while the TCGA dataset (*n* = 702) was used as an independent training cohort. Additionally, four GEO datasets—GSE4412 (*n* = 85), GSE13041 (*n* = 191), GSE43378 (*n* = 50), and GSE83300 (*n* = 50)—were obtained for further validation and evaluation of the predictive model.

**TABLE 1:** Clinical information of patients.

TCGA database	No. of patients (*n* = 702)	CGGA database	No. of patients (*n* = 1018)
Age		Age	
< 45	284	< 45	573
≥ 45	325	≥ 45	444
NA	93	NA	1
Gender		Gender	
Male	354	Male	601
Female	255	Female	417
NA	93	NA	0
WHO grade		WHO grade	
Grade II	216	Grade II	291
Grade III	241	Grade III	334
Grade IV	152	Grade IV	388
NA	93	NA	5
IDH mutation		IDH mutation	
Mutation	428	Mutant	531
Wildtype	234	Wildtype	435
NA	40	NA	52
1p/19q codeletion		1p/19q codeletion	
Codeletion	169	Codeletion	212
Non‐codeletion	495	Non‐codeletion	728
NA	38	NA	78
Subtypes		Subtypes	
Proneural	448	Proneural	379
Neural	52	Neural	296
Classical	160	Classical	191
Mesenchymal	42	Mesenchymal	152

### 2.2 Prognostic Value Analysis of ITPRIPL1

We first downloaded gene sets of four transcriptional subtypes of glioma from the GSEA platform (https://www.gsea‐msigdb.org/gsea/index.jsp) and assessed the expression profiles of these subtypes across all cohorts. Receiver operating characteristic (ROC) curve analysis was then performed to validate the specificity of the classification results. Next, univariate Cox regression analysis was conducted to identify variables significantly associated with prognosis (*p* < 0.05), which were subsequently included in a multivariate Cox regression model. Finally, Kaplan–Meier (K‐M) survival analysis was used to compare OS between high and low ITPRIPL1 expression groups.

### 2.3 Immune Infiltration Analysis

To evaluate the immune infiltration status of ITPRIPL1 in the glioma microenvironment, we obtained immune profiling results using the CIBERSORT algorithm from the TIMER platform and visualized the distribution of ITPRIPL1 expression across 22 immune cell types. Immune cell marker gene sets were retrieved from the TISIDB database (http://cis.hku.hk/TISIDB/), and single‐sample gene set enrichment analysis (ssGSEA) was performed to estimate the infiltration levels of 28 immune cell types in glioma patients. In addition, immune, stromal, and microenvironment scores were calculated for each patient using the xCell algorithm based on gene expression data. Pearson correlation analysis was then conducted to investigate the relationship between ITPRIPL1 expression and the expression of common immune checkpoint molecules.

### 2.4 Drug Sensitivity Analysis

Potential therapeutic agents associated with ITPRIPL1 expression were screened using the GSCA platform (https://guolab.wchscu.cn/GSCA). Expression and drug response data were downloaded from the GDSC (https://www.cancerrxgene.org/) database, along with expression data from CGGA and TCGA glioma patients. Drug sensitivity prediction was then performed using the oncoPredict R package.

### 2.5 Molecular Docking

The structure of ITPRIPL1 was retrieved from the UniProt database (https://www.uniprot.org/), and small‐molecule structures of candidate drugs were downloaded from the PubChem database (https://pubchem.ncbi.nlm.nih.gov/). Water molecules and heteroatoms were removed using PyMOL software. The processed protein and drug structures were submitted to the CB‐Dock2 platform (https://cadd.labshare.cn/cb‐dock2/php/index.php) for molecular docking analysis. Finally, the docking results were visualized using PyMOL. The molecular docking results calculated by CB‐Dock2 are presented in Table S1.

### 2.6 Functional Enrichment Analysis

Genes significantly correlated with ITPRIPL1 (threshold: *p* < 0.001, cor > 0.3) were identified through Pearson correlation analysis and subjected to Gene Ontology (GO) enrichment analysis. Immune‐related gene sets were downloaded from the Molecular Signatures Database (MSigDB, http://software.broadinstitute.org/gsea/msigdb/), and gene set variation analysis was performed to compute enrichment scores, followed by data visualization. Furthermore, Pearson correlation was used to calculate *p*‐values and correlation coefficients (*R*) between ITPRIPL1 and various immune‐related processes.

### 2.7 Construction of the Optimal Prognostic Model Using 98 Machine Learning Combinations

A recently proposed machine learning framework, Mime, enables the construction of optimal prognostic models for patients based on input variables and cohort data. This framework integrates 10 classical machine learning algorithms and four feature selection methods (Lasso, StepCox, CoxBoost, and RSF), resulting in a total of 98 parameter combinations. Model training was performed using *K*‐fold cross‐validation on the training set, and the optimal model was selected based on the maximization of the average concordance index (C‐index) across the validation cohorts. Therefore, using genes correlated with ITPRIPL1 and datasets from the training cohort (TCGA) and multiple validation cohorts (CGGA.array, CGGA.325, CGGA.693, CGGA.1018, GSE4412, GSE13041, GSE43378, and GSE83300), the Mime R package was used to construct the optimal prognostic model. The specific parameters of the 10 machine learning algorithms are presented in Table S2.

### 2.8 Evaluation of Model Performance

Patients were divided into high‐risk and low‐risk groups based on the median risk score calculated by the optimal model, and Kaplan–Meier survival curves were plotted. Time‐dependent ROC curve analysis was conducted using the Mime package to calculate the 1‐year, 2‐year, and 3‐year area under the ROC curve (AUC) values of the top 15 model combinations across all cohorts, thereby evaluating the predictive performance of the optimal model among the 98 combinations. Additionally, the Mime package includes 95 previously published glioma prognostic models (including LGG and GBM), allowing for comparison of hazard ratios (HRs), C‐index, and AUC values (1‐, 2‐, and 3‐year) between the best‐performing model in this study and existing models to assess its relative predictive power. The characteristics and coefficients of 95 published glioma models are presented in Table S3. To further determine the prognostic value of the optimal model, univariate Cox regression meta‐analysis was conducted across all cohorts, followed by multivariate Cox regression analysis incorporating known risk factors.

### 2.9 Construction and Validation of the Nomogram

Using data from the TCGA training set and the CGGA.1018 validation cohort, a nomogram was constructed with the rms R package to predict 1‐, 2‐, and 3‐year OS probabilities in glioma patients. The variables included WHO grade, age, 1p/19q codeletion status, IDH mutation status, and risk score. Calibration curves were plotted to assess the consistency and accuracy of the nomogram.

### 2.10 Statistical Analysis and Visualization

Statistical analysis and data visualization were primarily performed using R software (version 4.3.2, Windows), IBM SPSS Statistics (version 26, Windows), GraphPad Prism (version 9, Windows), PyMOL (version 3.1.4, Windows), and Adobe Illustrator 2024 (Windows). Various R packages were used for data visualization and analysis, including Mime, ggplot2, pheatmap, readxl, forestplot, GSVA, oncoPredict, ggpubr, survminer, survival, Matrix, foreach, clusterProfiler, limma, ggvenn, rms, Seurat, patchwork, and pROC. Pearson or Spearman correlation analyses were applied to assess correlations between two variables. Unpaired *t*‐tests were used to evaluate differences between two groups, and one‐way ANOVA was used for comparisons among more than two groups. A *p*‐value < 0.05 was considered statistically significant (*p < 0.05; **p < 0.01; ***p < 0.001; ****p < 0.0001; ns indicates no statistical difference).

## 3 Results

### 3.1 High ITPRIPL1 Expression Is Associated With More Malignant Tumors

**FIGURE 2:**
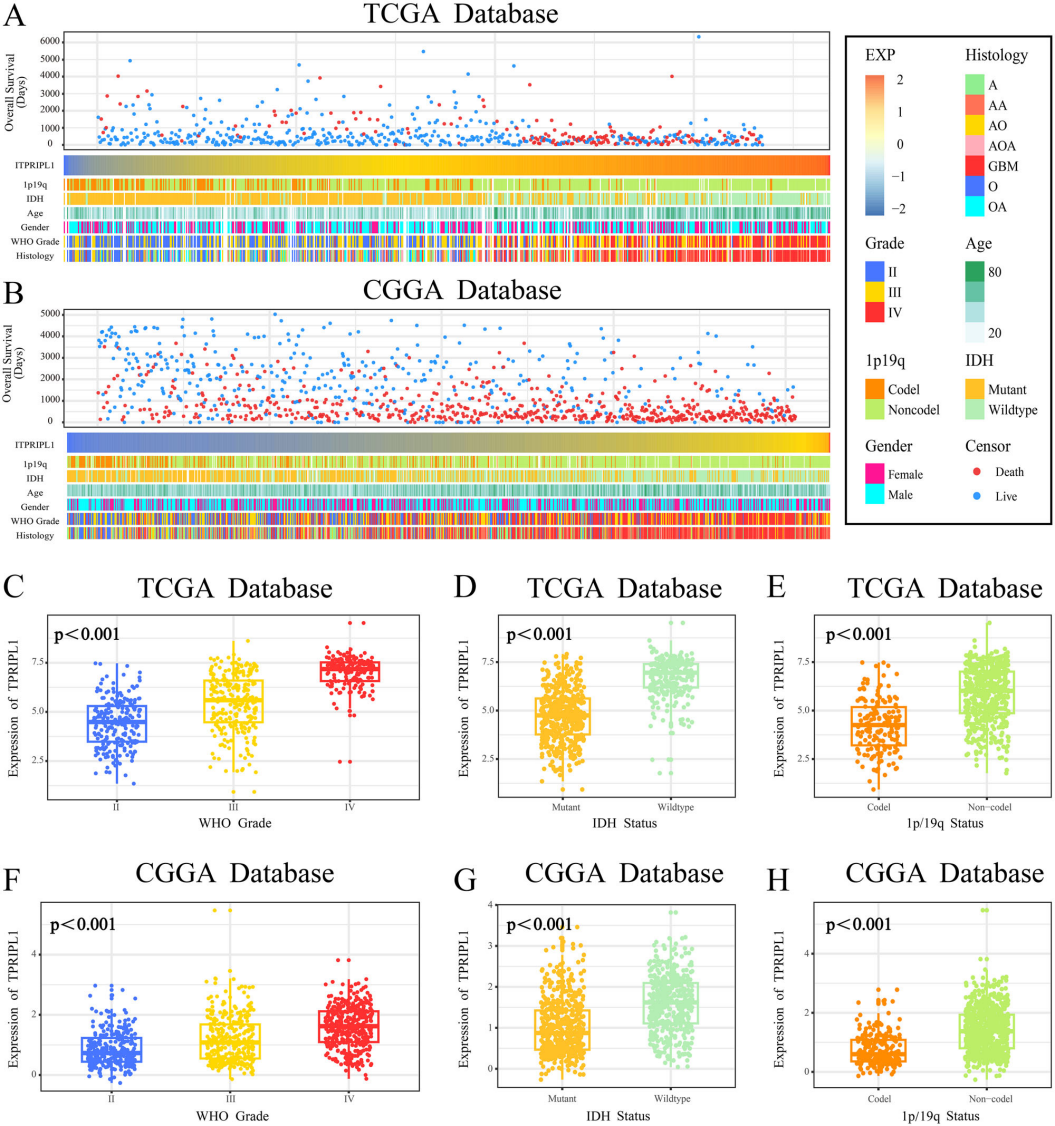
Association between ITPRIPL1 expression and clinicopathological features of glioma. (A and B) Overview of the correlation between ITPRIPL1 expression and glioma‐related clinicopathological features in the TCGA and CGGA cohorts. (C and F) ITPRIPL1 expression is significantly upregulated in patients with high‐grade glioma (one‐way ANOVA). (D and G) ITPRIPL1 expression is significantly higher in IDH wild‐type glioma patients (unpaired *t*‐test). (E and H) ITPRIPL1 expression is significantly increased in glioma patients without 1p/19q co‐deletion (unpaired *t*‐test).

Analysis using GEPIA and TIMER revealed that ITPRIPL1 is highly expressed in various tumor types compared to normal tissues, with a particularly significant upregulation observed in GBM (Figure S1). The expression level of ITPRIPL1 was associated with clinical and pathological features of patients. As ITPRIPL1 expression increased, there was an uneven distribution of 1p/19q codeletion status, IDH mutation status, WHO grade, and histological diagnosis in both the CGGA and TCGA datasets (Figure 2A,B). In the TCGA dataset, subgroup analyses indicated that ITPRIPL1 expression was higher in high‐grade glioma (Figure 2C), IDH wild‐type glioma (Figure 2D), and glioma without 1p/19q codeletion (Figure 2E). Validation in the CGGA dataset yielded results highly consistent with those in the TCGA training cohort (Figure 2F–H). Furthermore, an increase in ITPRIPL1 expression was associated with a marked reduction in OS among glioma patients (Figure 2A,B). These findings suggest that high ITPRIPL1 expression is linked to greater malignancy and poorer prognosis in glioma.

### 3.2 ITPRIPL1 Serves as an Independent Prognostic Factor for OS in Glioma Patients

**FIGURE 3:**
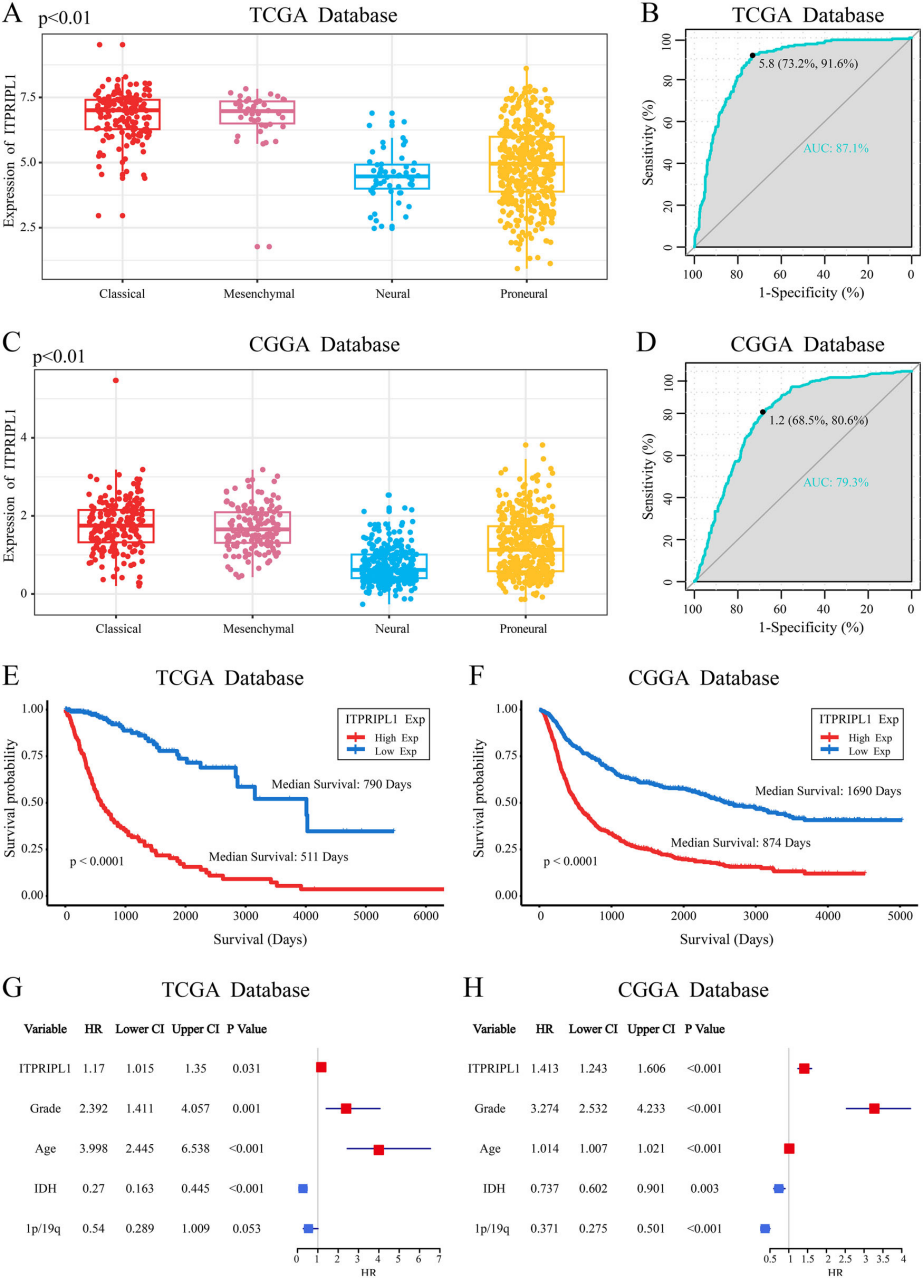
Prognostic significance of ITPRIPL1 in glioma. (A and C) Enrichment of ITPRIPL1 across different glioma subtypes in the TCGA and CGGA cohorts (one‐way ANOVA). (B and D) ROC curves validating the specificity of ITPRIPL1 enrichment across glioma subtypes in TCGA and CGGA datasets. (E and F) Kaplan–Meier survival analyses of ITPRIPL1 expression in TCGA and CGGA datasets. (G and H) Multivariate Cox regression forest plots for ITPRIPL1 in the TCGA and CGGA cohorts.

Glioma transcriptomic subtypes have been widely recognized globally, with classical and mesenchymal subtypes typically associated with poorer outcomes (Verhaak et al. 2010). We examined the distribution of ITPRIPL1 across these subtypes and found it to be enriched in classical and mesenchymal gliomas (Figure 3A,C). In addition, ROC curve analysis showed that the AUC value of ITPRIPL1 was 91.6% in the TCGA dataset and 80.6% in the CGGA dataset (Figure 3B,D), further confirming the specificity of the findings. Kaplan–Meier and Cox proportional hazard analyses based on the TCGA and CGGA datasets reinforced the prognostic significance of ITPRIPL1 in glioma patients. In the TCGA cohort, patients with high ITPRIPL1 expression had a significantly shorter OS (median survival: 511 days) compared to those with low expression (median survival: 790 days) (Figure 3E). Validation using the CGGA dataset corroborated these results (Figure 3F). Cox regression analysis demonstrated that ITPRIPL1 expression was an independent prognostic factor and showed no significant association with WHO grade, age at diagnosis, IDH mutation, or 1p/19q codeletion status (Figure 3G,H).

### 3.3 Immune Infiltration Analysis

**FIGURE 4:**
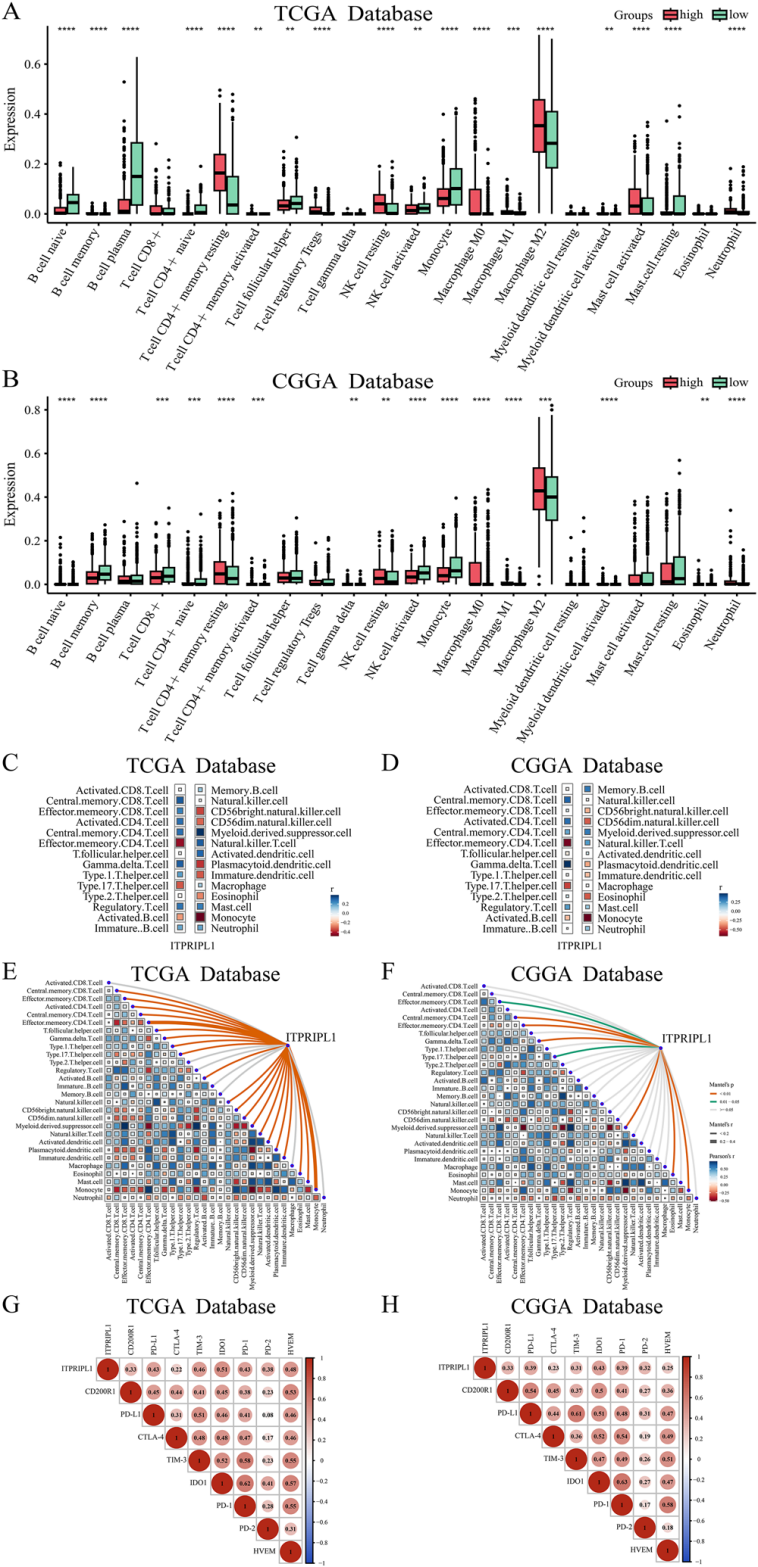
Impact of ITPRIPL1 expression on immune infiltration in glioma. (A and B) Correlation between ITPRIPL1 expression and 22 immune cell types calculated by the CIBERSORT algorithm in the TCGA and CGGA cohorts. (C and F) Correlation between ITPRIPL1 expression and 28 immune cell types estimated by ssGSEA in the TCGA and CGGA cohorts. (G and H) Pearson correlation matrix showing the association between ITPRIPL1 and immune checkpoint genes in the TCGA and CGGA datasets.

Using the CIBERSORT algorithm to estimate the infiltration levels of 22 immune cell types, we found that high ITPRIPL1 expression in the TCGA dataset was associated with increased infiltration of M2 macrophages (Figure 4A). This finding was further validated using the CGGA dataset (Figure 4B). ssGSEA analysis revealed a significant negative correlation between high ITPRIPL1 expression and both effector memory CD4⁺ T cells and type 17 T helper cells in the TCGA training cohort and CGGA validation cohort (Figure 4C,D), which was further confirmed by Pearson correlation analysis (Figure 4E,F). The xCell algorithm indicated that ITPRIPL1 expression was positively correlated with immune score, microenvironment score, and stromal score (Figure S2). Additionally, we investigated the relationship between ITPRIPL1 and known inhibitory immune checkpoints, including CD200R1, PD‐L1, CTLA‐4, TIM‐3, IDO1, PD‐1, PD‐2, and HVEM in both the TCGA and CGGA datasets (Figure 4G,H). The results showed positive correlations between ITPRIPL1 and these immunosuppressive checkpoints, suggesting that ITPRIPL1 may contribute to immune evasion in glioma.

### 3.4 Drug Screening and Target Prediction Analysis

**FIGURE 5:**
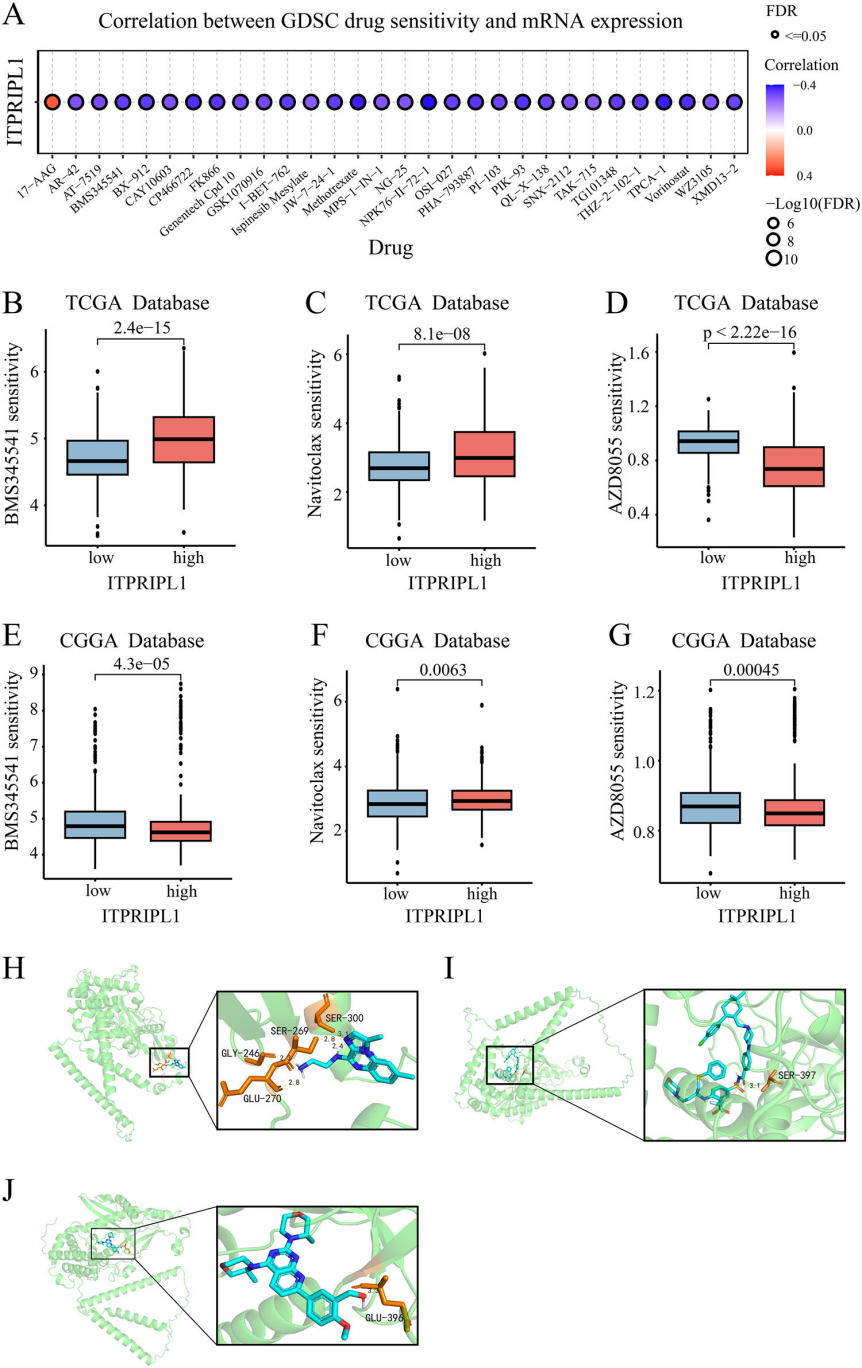
Drug screening and target prediction analysis of ITPRIPL1. (A) Top 30 drugs most significantly associated with ITPRIPL1 expression in the GDSC database. (B–D) Correlation analysis between ITPRIPL1 expression and drug sensitivity in the TCGA cohort. (E–G) Correlation analysis between ITPRIPL1 expression and drug sensitivity in the CGGA cohort. (H–J) Molecular docking analysis of ITPRIPL1 protein with BMS‐345541, Navitoclax, and AZD8055.

We first used the GDSC database to identify the top 30 antitumor drugs most strongly correlated with ITPRIPL1 expression (Figure 5A). Subsequently, drug sensitivity analysis based on the TCGA dataset revealed that ITPRIPL1 expression was positively correlated with BMS‐345541 and Navitoclax, but negatively correlated with AZD8055 (Figure 5B–D). However, in the CGGA dataset, BMS‐345541 expression showed a negative correlation with ITPRIPL1 (Figure 5E–G). To further explore the interaction between ITPRIPL1 and candidate drugs, we conducted molecular docking analysis. The results showed that BMS‐345541, Navitoclax, and AZD8055 all had the potential to bind to the ITPRIPL1 protein. Among them, Navitoclax had the strongest predicted binding energy (−10.6 kcal/mol), which was significantly better than AZD8055 (−9.4 kcal/mol) and BMS‐345541 (−6.5 kcal/mol) (Figure 5H–J; Table S1).

### 3.5 Correlation between ITPRIPL1 and the Negative Regulation of T Cells

**FIGURE 6:**
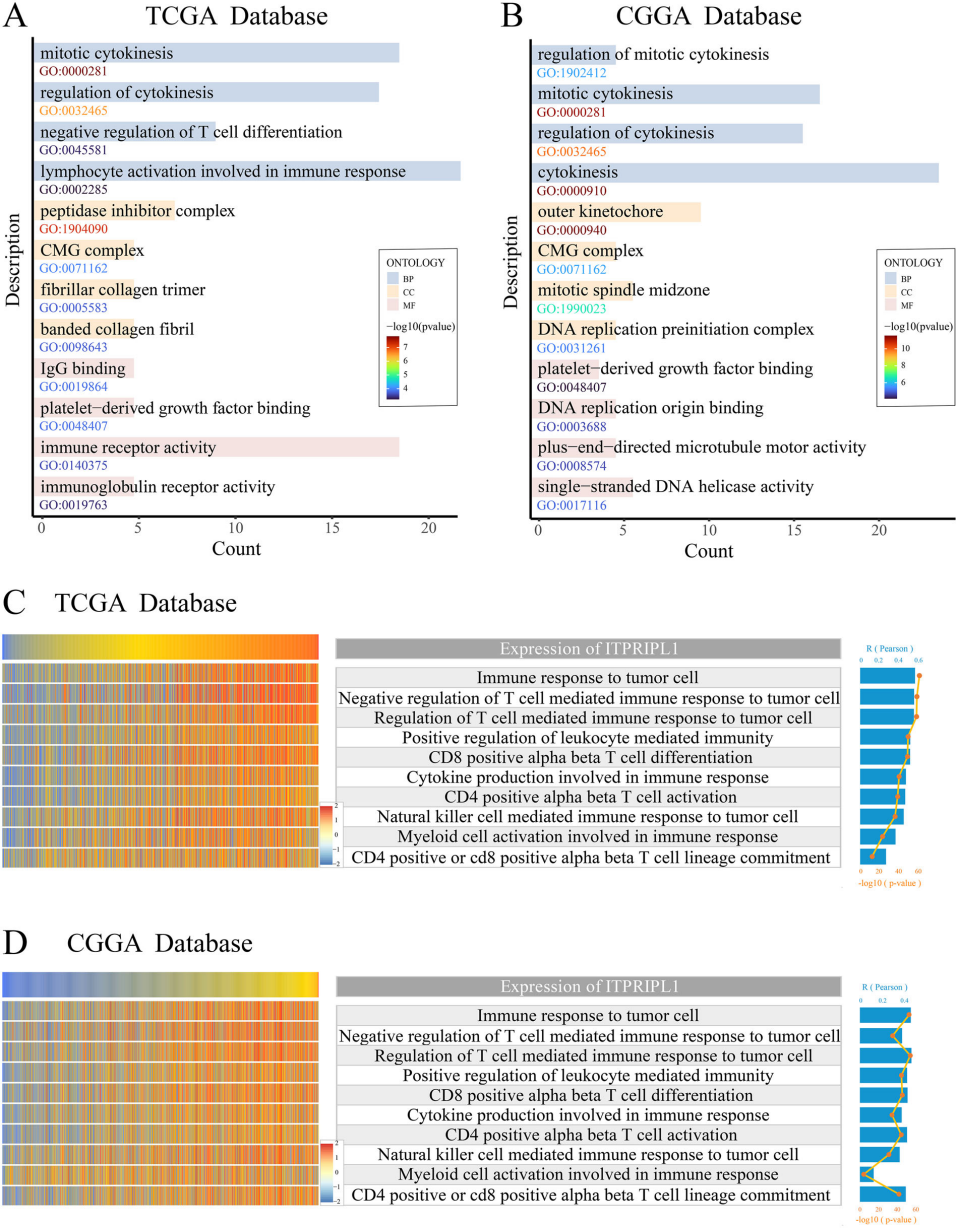
Association between ITPRIPL1 and negative regulation of T cell function. (A and B) GO enrichment analysis of ITPRIPL1‐related genes in the TCGA and CGGA datasets. (C and D) Heatmaps showing the association between ITPRIPL1 expression and immune functional enrichment scores in TCGA and CGGA cohorts.

In the TCGA dataset, biological processes associated with ITPRIPL1 were mainly enriched in immune regulation, cell division, and the cell cycle; the most enriched cellular component was the CMG complex; and molecular functions were primarily related to immune receptor activity and DNA binding (Figure 6A). Similar results were observed in the CGGA dataset (Figure 6B). To further validate the potential role of ITPRIPL1 in immune responses and regulation in glioma, we performed gene set variation analysis to calculate the enrichment scores of immune‐related processes in both the TCGA and CGGA datasets. The results revealed that ITPRIPL1 expression was positively correlated with most immune functions, particularly with the “Negative regulation of T cell mediated immune responses to tumor cells” in the TCGA dataset (Figure 6C). This trend was similarly confirmed in the CGGA dataset (Figure 6D). These findings suggest that ITPRIPL1 may be involved in the negative regulation of T cells within the glioma immune microenvironment.

### 3.6 Construction of an ITPRIPL1‐Associated Prognostic Model Using Mime

**FIGURE 7:**
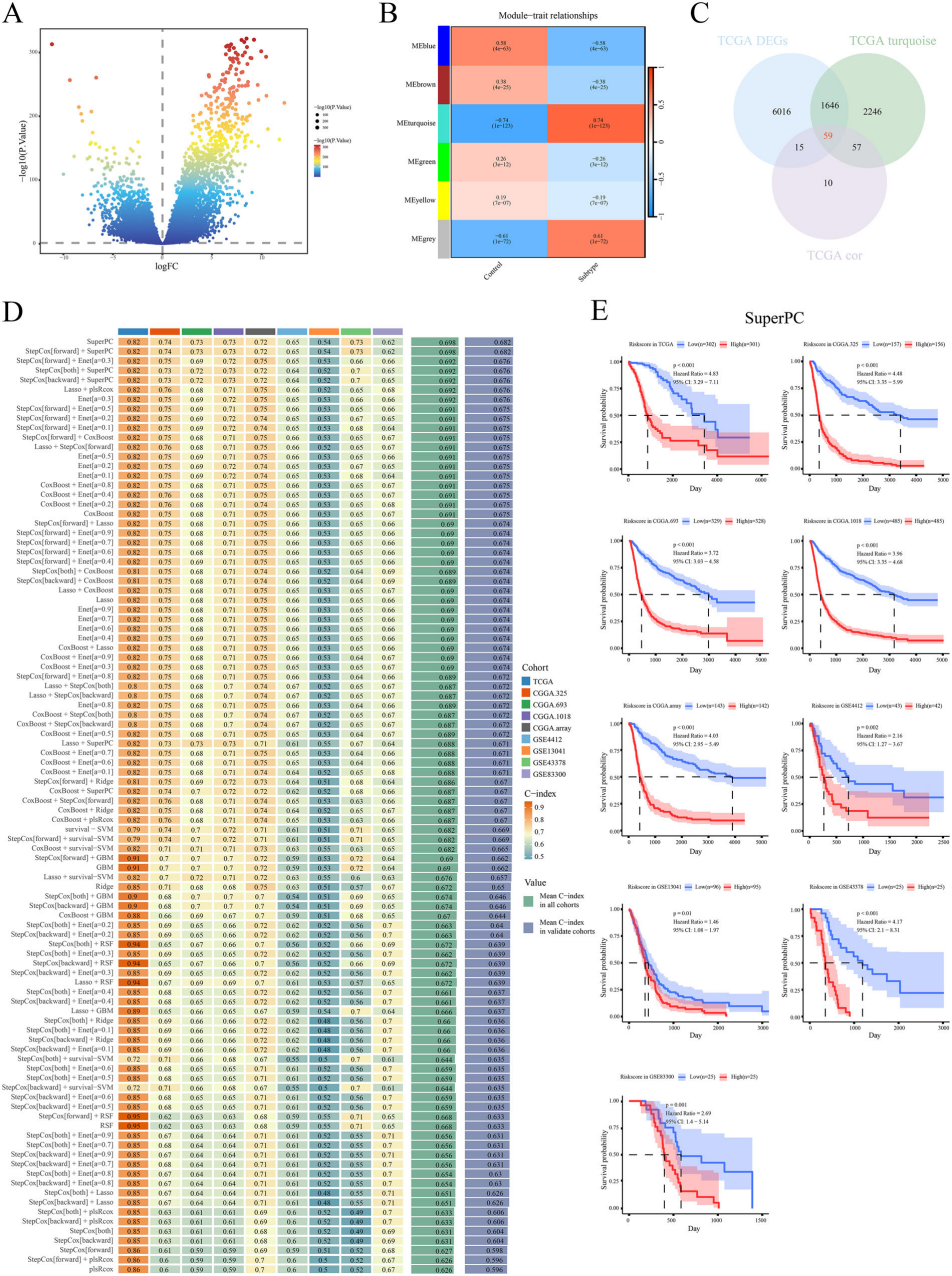
Construction of a prognostic model based on ITPRIPL1‐related features. (A) Volcano plot of differentially expressed genes in the TCGA dataset. (B) WGCNA analysis of the TCGA dataset. (C) Venn diagram showing the overlap among differentially expressed genes, WGCNA modules, and Pearson correlation analysis in the TCGA cohort. (D) Ranking of C‐index values for each model in the TCGA, CGGA, and GEO datasets (ordered by average C‐index in validation cohorts). (E) Kaplan–Meier survival curves based on risk scores in the TCGA, CGGA, and GEO cohorts.

A total of 7736 differentially expressed genes were identified in the TCGA dataset (Figure 7A). Through WGCNA analysis, 4008 ITPRIPL1‐related genes were identified (Figure 7B). An additional 141 genes were selected based on Pearson correlation analysis. The intersection of these three gene sets yielded 59 co‐expressed genes (Figure 7C). Using the Mime machine learning framework, the model with the highest average C‐index was identified as SuperPC (Figure 7D). Based on the risk scores calculated from the machine learning model, glioma patients were stratified into high‐risk and low‐risk groups using the median score as the cutoff. Survival analysis across all cohorts demonstrated that patients in the high‐risk group had significantly worse outcomes (Figure 7E).

### 3.7 Performance Evaluation of SuperPC

**FIGURE 8:**
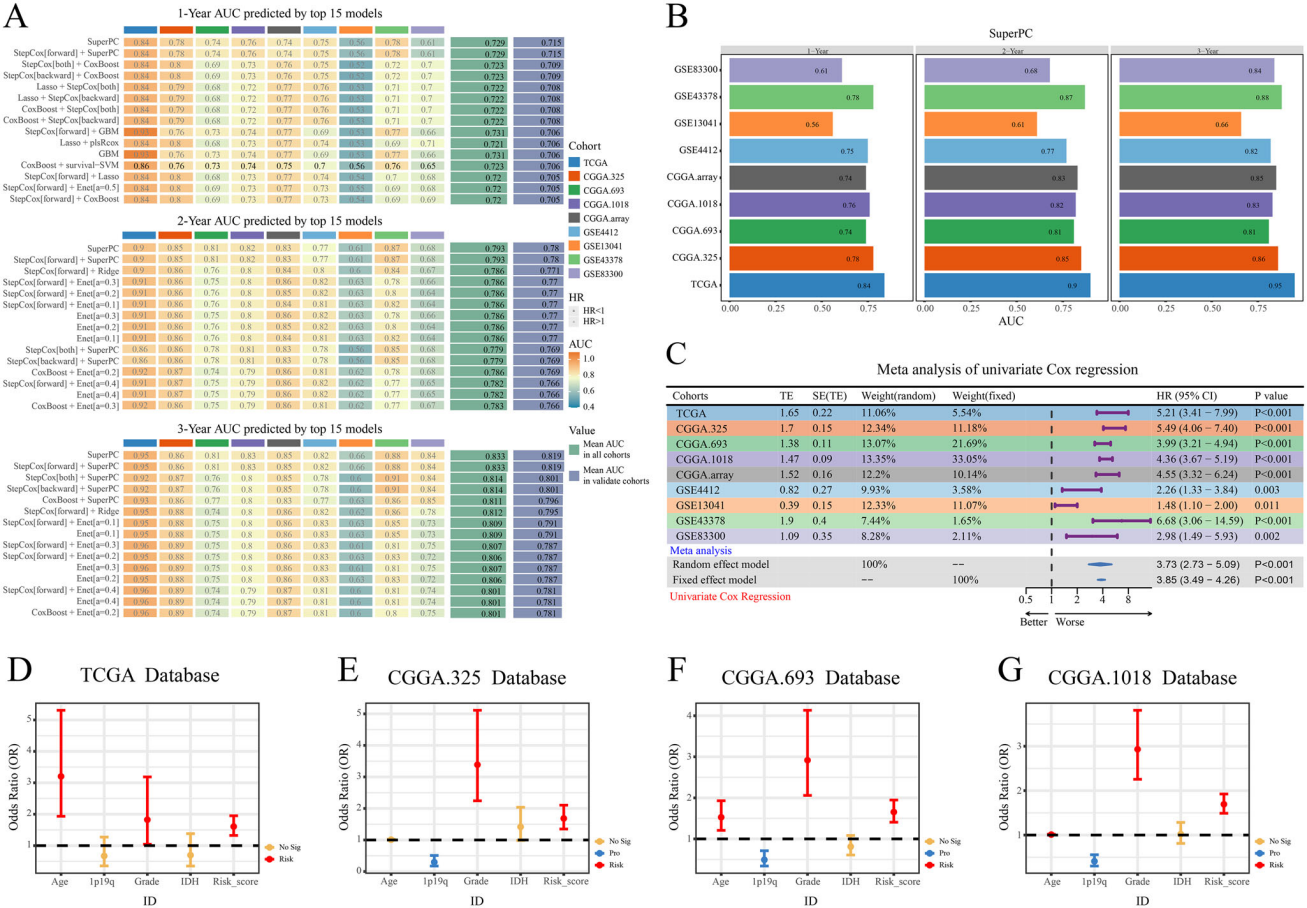
Evaluation of prognostic model performance. (A) AUC values at 1, 2, and 3 years for the top 15 models in the TCGA, CGGA, and GEO datasets. (B) AUC performance of the SuperPC model at 1, 2, and 3 years in the TCGA, CGGA, and GEO datasets. (C) Meta‐analysis of univariate Cox regression results across the TCGA, CGGA, and GEO cohorts. (D–G) Forest plots of multivariate Cox regression analysis in the TCGA and CGGA cohorts.

As the AUC is another critical indicator for evaluating model performance, we assessed SuperPC using ROC curve analysis via the Mime framework. In both the training cohort (TCGA) and validation cohorts (CGGA and GEO), the ROC curves for 1‐, 2‐, and 3‐year survival predictions showed strong predictive performance (Figure 8A,B). To evaluate the prognostic value of SuperPC, we performed a univariate Cox regression meta‐analysis using Mime. The results revealed that the risk score computed by SuperPC was a significant risk factor for glioma (Figure 8C). Furthermore, multivariate Cox regression analysis incorporating known glioma biomarkers showed that the SuperPC score remained an independent prognostic factor, unrelated to diagnostic age, WHO grade, IDH mutation status, or 1p/19q codeletion status (Figure 8D–G). These findings indicate that the SuperPC model built via Mime demonstrates high accuracy in prognostic prediction.

### 3.8 Comparison of SuperPC With 95 Published Models

**FIGURE 9:**
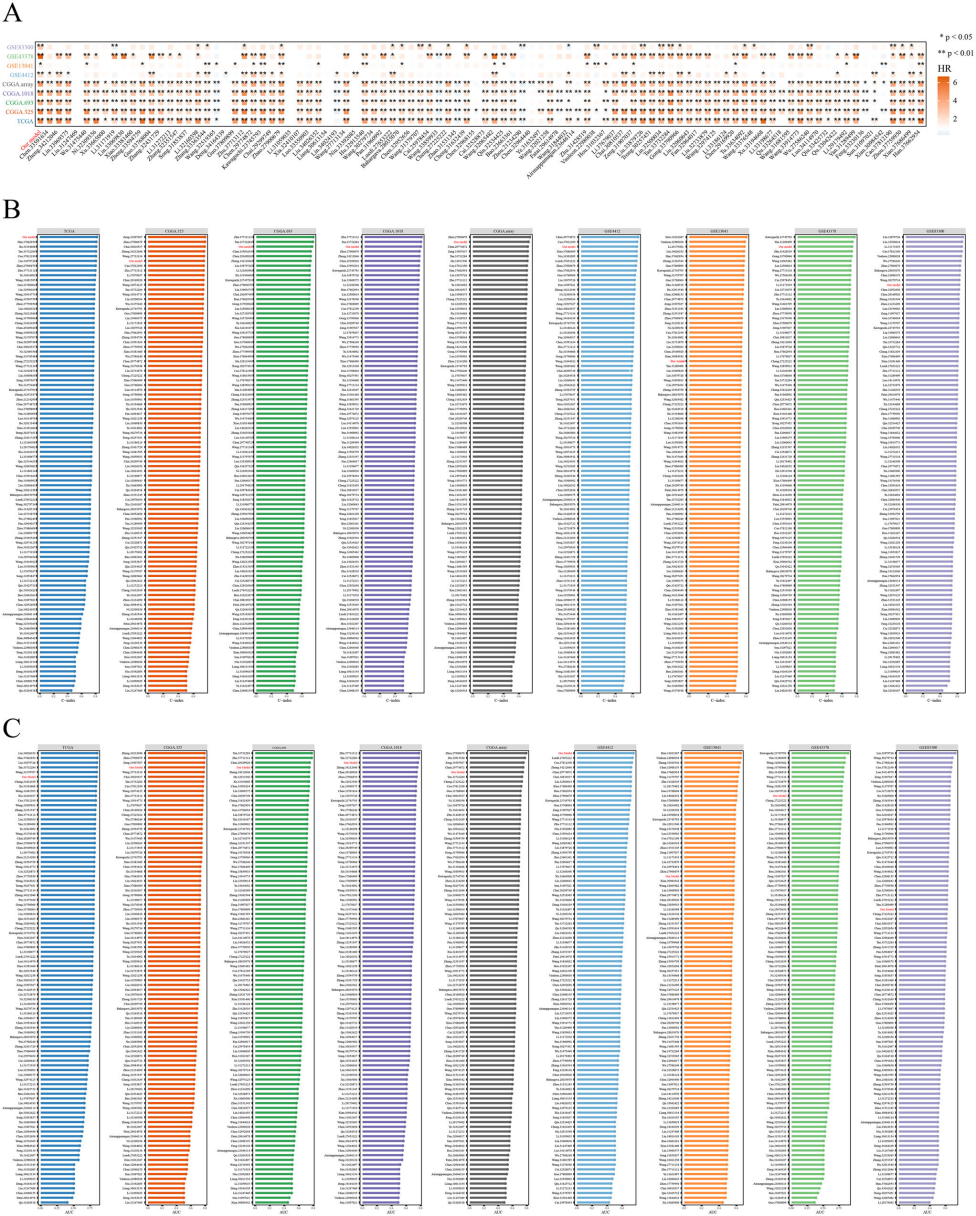
Comparison of the optimal model with previously published glioma models. (A) Comparison of hazard ratios (HRs) between the SuperPC model and 95 published glioma models. (B) Comparison of C‐index values between the SuperPC model and 95 published models. (C) Comparison of 1‐year AUC values between the SuperPC model and 95 published models.

In recent years, machine learning–based prognostic and predictive models have been widely applied in glioma research. To comprehensively compare SuperPC with other published models, we utilized 95 previously established glioma models integrated within Mime and conducted univariate Cox regression analyses across the TCGA, CGGA, and GEO datasets to assess their prognostic relevance. The results showed that SuperPC exhibited the most significant prognostic correlation among all models (Figure 9A). Additionally, SuperPC ranked among the top in terms of concordance index (C‐index) across datasets (Figure 9B). Similarly, the AUC values for 1‐, 2‐, and 3‐year survival predictions were significantly higher for SuperPC compared to other models (Figure 9C; Figure S3). These findings demonstrate that SuperPC possesses superior accuracy and robustness in predicting glioma patient prognosis.

### 3.9 Immune Infiltration Analysis of SuperPC

**FIGURE 10:**
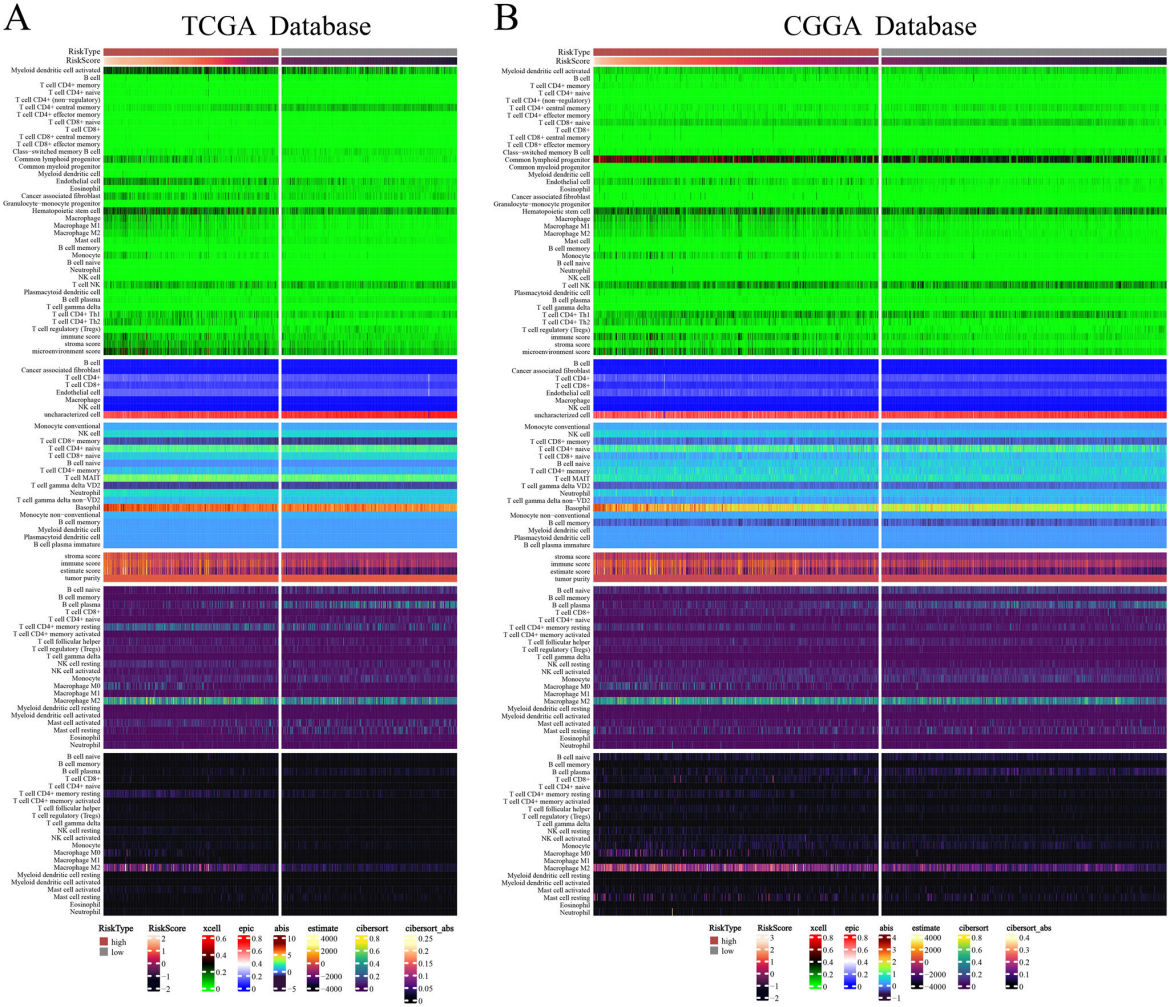
Correlation between risk score and immune characteristics. (A) Correlation between risk score calculated by the SuperPC model and tumor microenvironment features derived from multiple deconvolution algorithms in the TCGA cohort. (B) Correlation between SuperPC‐derived risk scores and tumor microenvironment features in the CGGA cohort.

Immune infiltration and TME analysis conducted using the immunedeconv and IOBR tools within the Mime framework revealed that the high‐risk group, as defined by SuperPC, exhibited significantly higher immune infiltration scores than the low‐risk group in both TCGA and CGGA cohorts. Additionally, CIBERSORT analysis showed that the high‐risk group was predominantly enriched in M2 macrophages (Figure 10A,B).

### 3.10 Construction of a Nomogram

**FIGURE 11:**
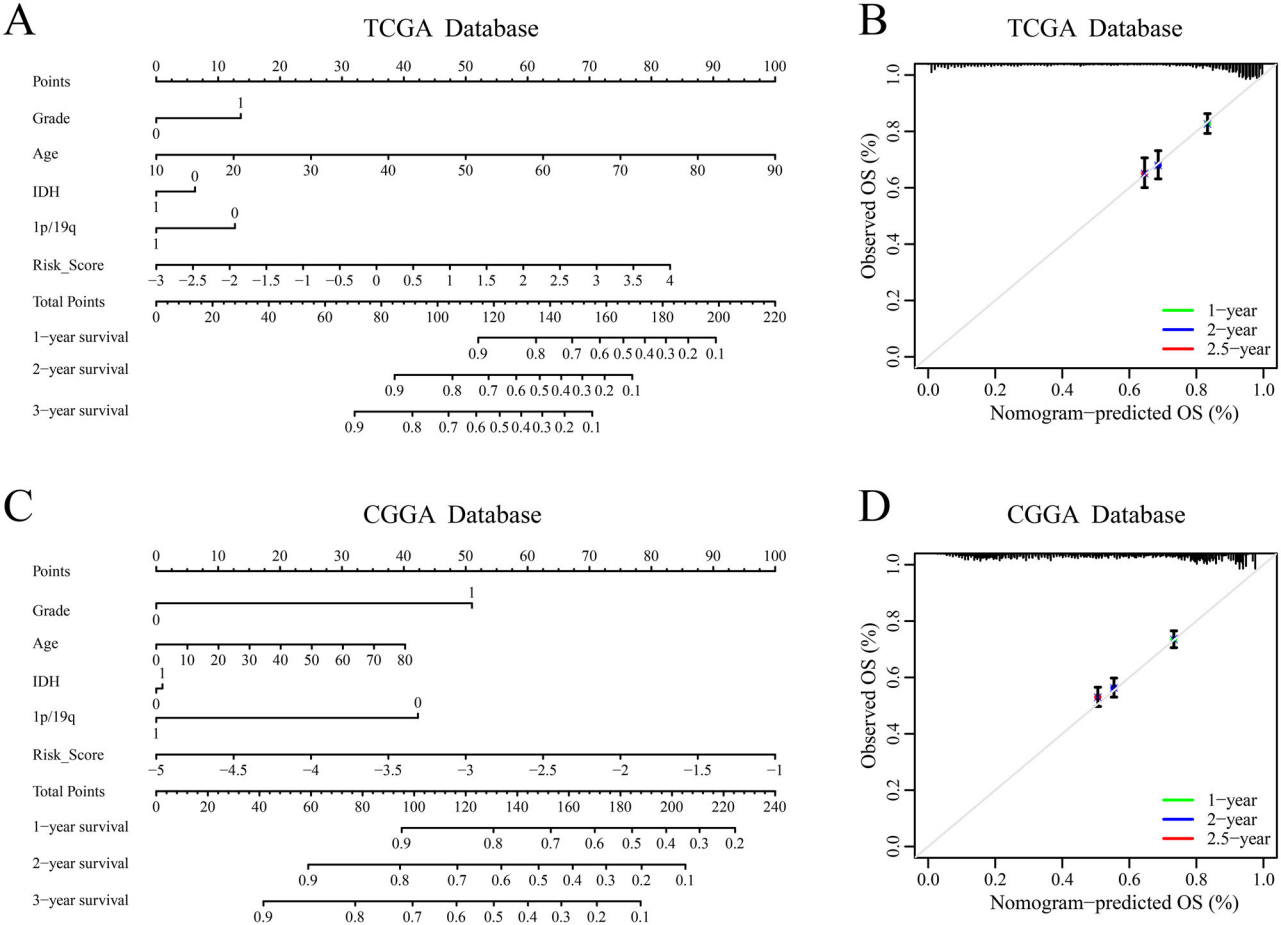
Construction and validation of nomograms. (A and B) Nomograms predicting survival probability of glioma patients in the TCGA and CGGA cohorts. (C and D) Calibration plots of the nomograms in the TCGA and CGGA cohorts.

We constructed a nomogram to predict 1‐, 2‐, and 3‐year survival probabilities for glioma patients by incorporating WHO grade, age, 1p/19q codeletion status, IDH mutation status, and SuperPC‐derived risk score (Figure 11A,C). Calibration curves demonstrated a strong consistency between predicted and observed survival outcomes (Figure 11B,D). Collectively, these results suggest that the nomogram provides accurate prognostic predictions for glioma patients.

## 4 Discussion

Glioma is one of the most common and aggressive malignant tumors of the central nervous system (Price et al. 2024). Advances in immunotherapy have significantly accelerated research into the molecular mechanisms of glioma and the development of clinical treatments (Yasinjan et al. 2023). However, the heterogeneity and immunosuppressive nature of the glioma microenvironment remain major barriers to effective preoperative diagnosis, disease monitoring, and therapeutic efficacy (Yasinjan et al. 2023). Therefore, in‐depth investigation of the glioma immune microenvironment and identification of novel immunotherapeutic targets have become urgent priorities.

A recent study demonstrated that ITPRIPL1, as a novel inhibitory ligand of CD3ε, enables tumors to evade immune surveillance by directly targeting key signaling pathways involved in T cell activation. Blocking this pathway may offer a promising therapeutic approach for tumors with resistance to PD‐1/PD‐L1 blockade or those exhibiting low immune infiltration (Deng et al. 2024). In our study, we found that high expression of ITPRIPL1 is positively correlated with glioma malignancy and is associated with poor prognosis in glioma patients. GO and GSVA analyses further revealed that the expression level of ITPRIPL1 was negatively correlated with T cell–mediated antitumor immune responses, suggesting that ITPRIPL1 may promote glioma progression by suppressing T cell activity and modulating the TME. An increasing body of evidence highlights the crucial role of the TME in glioma progression, particularly the immunosuppressive regulation of T cell function within the TME (Read et al. 2024; Tataranu et al. 2024). Immune infiltration analysis showed that ITPRIPL1 expression was negatively associated with the infiltration of effector memory CD4⁺ T cells and Th17 cells, but positively associated with M2 macrophage infiltration. Effector memory CD4⁺ T cells are a critical T cell subset that persist in the TME and, upon IL‐12 stimulation, secrete large amounts of IFN‐γ to enhance the recruitment of immune cells such as macrophages and dendritic cells, thereby amplifying antitumor responses (Broderick et al. 2005).Th17 cells, which differentiate from naïve CD4⁺ T cells in response to specific antigens, play a dual role in tumor regulation by promoting both antitumor immunity through cytokine secretion (e.g., IL‐17) and tumor progression via angiogenesis and extracellular matrix remodeling (Wu et al. 2021). Furthermore, tumor‐associated macrophages (TAMs), particularly the M2 subtype, are highly enriched in the glioma microenvironment and contribute to malignancy by secreting pro‐tumorigenic factors such as IL‐10, TGF‐β, CCL22, and CCL17 (Khan et al. 2023; Zhang et al. 2021). M2 macrophages also promote tumor cell growth and immune evasion through metabolic reprogramming (Luo et al. 2020; Xiang and Miao 2021; Zang et al. 2019). These findings collectively suggest that ITPRIPL1 may facilitate glioma immune evasion by suppressing T cell activity and enhancing the pro‐tumor functions of M2 macrophages.

This study also found that ITPRIPL1 is positively correlated with common immune checkpoints such as PD‐1 and IDO1. In the glioma microenvironment, PD‐1 is widely expressed on T cells, B cells, TAMs, myeloid‐derived suppressor cells, and natural killer (NK) cells. The binding of PD‐1 to its ligand PD‐L1 inhibits T cell activation and proliferation, induces T cell apoptosis, and thereby enables tumor cells to evade immune surveillance (Shu and Li 2020). In addition, PD‐1 attenuates T cell activation and function by recruiting SHP‐1 and SHP‐2 phosphatases to inhibit key molecules in the TCR signaling pathway (Stanford et al. 2012). Kleffel et al. further demonstrated that PD‐1 expressed in tumor cells can activate the NF‐κB signaling pathway, thereby promoting tumor growth (Kleffel et al. 2015). IDO1 is the main subtype of the IDO family, and its overexpression has been associated with poor prognosis in glioma by suppressing the function of cytotoxic T lymphocytes and NK cells (Cheong et al. 2018). IDO1 expression is induced by inflammatory cytokines such as IFN‐γ and TNF‐α, and is transcriptionally regulated through the activation of signaling pathways including JAK/STAT and NF‐κB, leading to tryptophan metabolism disruption and the formation of an immunosuppressive microenvironment (Zhou et al. 2025). Di et al. confirmed that tumor cells can upregulate these immune checkpoints to escape immune surveillance. These findings suggest that ITPRIPL1 may resemble immune checkpoints in promoting tumorigenesis and progression by suppressing T cell function within the TME.

Drug sensitivity analysis revealed that the expression level of ITPRIPL1 was associated with sensitivity to Navitoclax, BMS‐345541, and AZD8055. Molecular docking results indicated that these sensitive drugs can directly bind to the ITPRIPL1 protein, suggesting that ITPRIPL1 may serve as a potential therapeutic target in glioma. Previous studies have demonstrated the potential of Navitoclax in glioma treatment, particularly when used in combination with the PI3K inhibitor GDC‐0941, 2‐deoxy‐D‐glucose (2‐DG), or metformin, which enhances its therapeutic efficacy (Levesley et al. 2013; Pareja et al. 2014). AZD8055, when combined with a CD40 agonistic antibody, has been shown to promote CD8⁺ T cell and NK cell infiltration and activation within the tumor site, thereby enhancing immunotherapeutic efficacy through remodeling of the tumor immune microenvironment (Jiang et al. 2011). In breast cancer and melanoma, BMS‐345541 exerts antitumor effects by inhibiting the NF‐κB signaling pathway (Battula et al. 2017; Yang et al. 2006). Recent studies have underscored the importance of genetic mutations and epigenetic regulation in tumor therapy resistance. For instance, mutations in IDH1/2 play a pivotal role in glioma resistance to treatment by producing the oncometabolite 2‐hydroxyglutarate (2‐HG), which inhibits TET DNA demethylases and JmjC domain‐containing histone demethylases, leading to global DNA hypermethylation and abnormal histone modifications, thereby promoting resistance to radiotherapy and chemotherapy (Guo et al. 2011). Notably, our results showed an opposite trend for BMS‐345541 between the TCGA and CGGA datasets. To investigate the possible reason for this opposite correlation, we found that the IDH mutation rate in the TCGA cohort (64.7%) is significantly higher than that in the CGGA cohort (55.0%), and we further compared the correlation between ITPRIPL1 and BMS‐345541 under different IDH statuses. The results showed that ITPRIPL1 was negatively correlated with BMS‐345541 under both IDH statuses, and this correlation was more significant in the TCGA cohort (Figure S4). To further explore the mechanism by which IDH status modulates the effect of BMS‐345541, we obtained the NF‐κB pathway gene set from the MSigDB database, calculated gene set enrichment scores using GSVA, and compared the correlation between BMS‐345541 and the NF‐κB pathway under different IDH statuses. The results indicated that BMS‐345541 exerted a stronger inhibitory effect on the NF‐κB pathway in the IDH‐mutant state, thereby playing an anticancer role (Figure S4). We speculate that this difference may be related to the variation in IDH mutation rates between the two cohorts and the epigenetic regulatory effects of IDH status on NF‐κB pathway activity. In addition, sample heterogeneity and technical differences may also partly contribute to the inconsistency of the results. Further clinical studies are needed to comprehensively evaluate these factors to develop more precise therapeutic strategies.

There are still some limitations in our study. First, although immunotherapy has brought new prospects to cancer treatment, glioma—especially GBM—exhibit high heterogeneity and a complex immune microenvironment. These features necessitate more in‐depth investigations, particularly in relation to therapeutic response. Second, this study mainly relied on data from public databases and lacked experimental validation. Future work should include in vitro and in vivo experiments to further elucidate the biological functions and underlying mechanisms of ITPRIPL1 in glioma.

## 5 Conclusion

This study reveals a dual biological role of ITPRIPL1 in glioma. On one hand, it contributes to the formation of an immunosuppressive TME by negatively regulating T cell–mediated immune responses. On the other hand, it accelerates malignant progression by compromising the efficacy of antitumor agents. These two mechanisms collectively lead to poorer clinical outcomes in patients. Therefore, ITPRIPL1 not only holds promise as a novel prognostic biomarker for glioma, but also emerges as a potential therapeutic target in the context of immunotherapy.

## Author Contributions

**Zou Xiaoyun**: formal analysis, validation, visualization, writing – original draft, methodology. **Ye Wenhao**: validation, writing – original draft, methodology. **Wu Huan**: data curation, writing – original draft, validation. **Yang Yuanyuan**: data curation, writing – original draft, validation. **Liu Changqing**: writing – original draft, validation, data curation. **Wen Hebao**: funding acquisition, project administration, supervision, writing – review and editing. **Ma Caiyun**: project administration, resources, supervision, writing – review and editing.

## Conflicts of Interest

The authors declare no conflicts of interest.

## Peer Review

The peer review history for this article is available at https://publons.com/publon/10.1002/brb3.70762

## Ethics Statement

The authors have nothing to report.

## Supporting information




**Supporting Fig.1‐fig.3**: brb370762‐sup‐0001‐SuppMat.docx

**Supporting fig.4**: brb370762‐sup‐0002‐SuppMat.docx

**Supporting Table 1**: brb370762‐sup‐0003‐tableS1.xlsx

**Supporting Table 2**: brb370762‐sup‐0004‐tableS2.xlsx

**Supporting Table 3**: brb370762‐sup‐0005‐tableS3.xlsx

## Data Availability

The datasets that were analyzed in this study are not publicly available. Further inquiries can be directed to the corresponding author.
