# Supplementary material for: Immune Evasion Mechanism Mediated by ITPRIPL1 and Its Prognostic Implications in Glioma
Source: Brain Behav. 2025 Aug 12;15(8):e70762. doi: 10.1002/brb3.70762 (PMC12340539; doi:10.1002/brb3.70762)
Supplement: Supplementary file 1 — Supporting Fig.1‐fig.3: brb370762‐sup‐0001‐SuppMat.docx [file BRB3-15-e70762-s002.docx]

***Supporting Information***

## Supplementary Figures

***
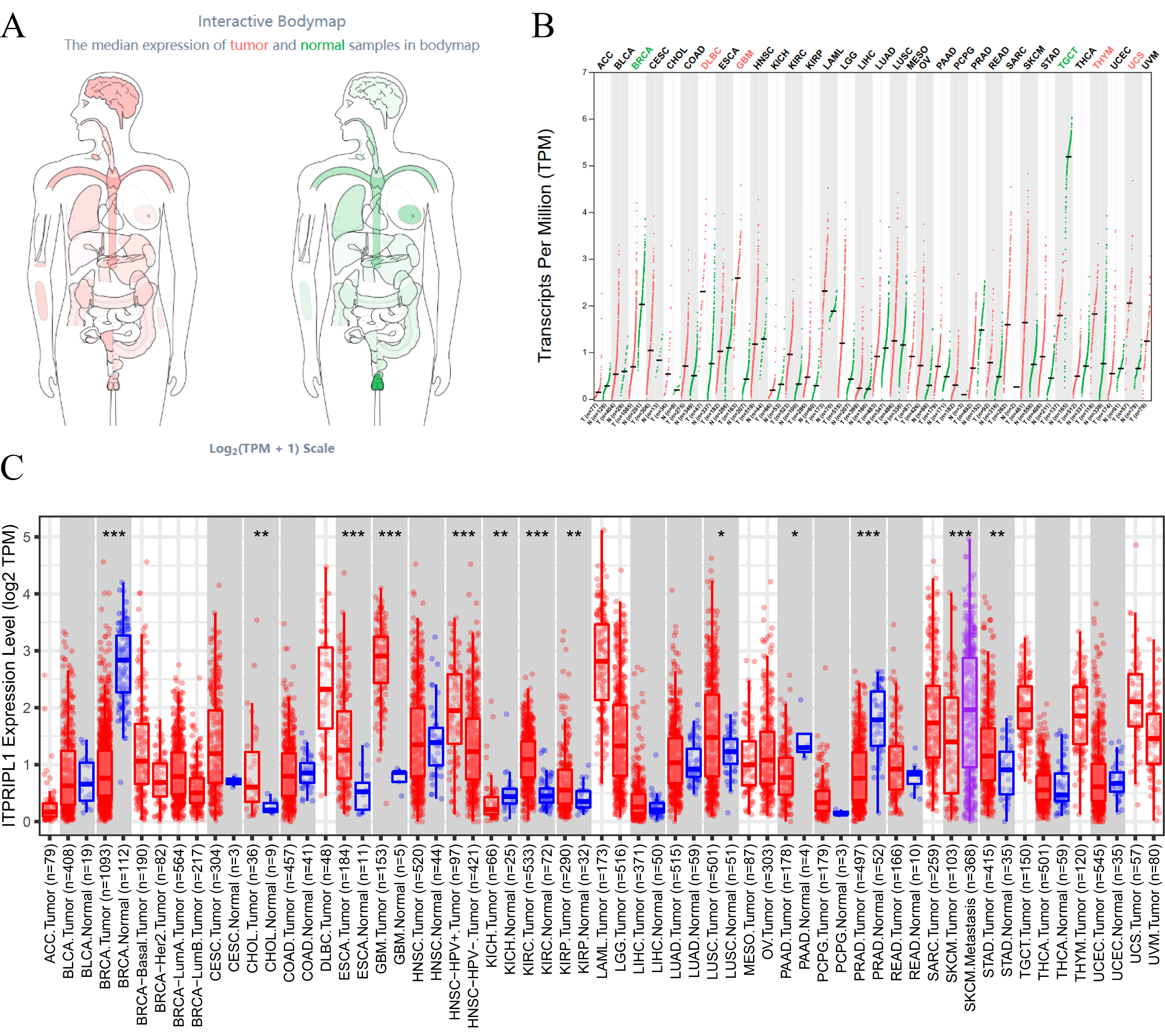
***

**Supplementary Figure 1. Elevated expression of ITPRIPL1 in glioma.** (A) Distribution of ITPRIPL1 expression in human tumor and normal tissues. (B-C) Expression levels of ITPRIPL1 in various tumor types and their corresponding normal tissues.

***
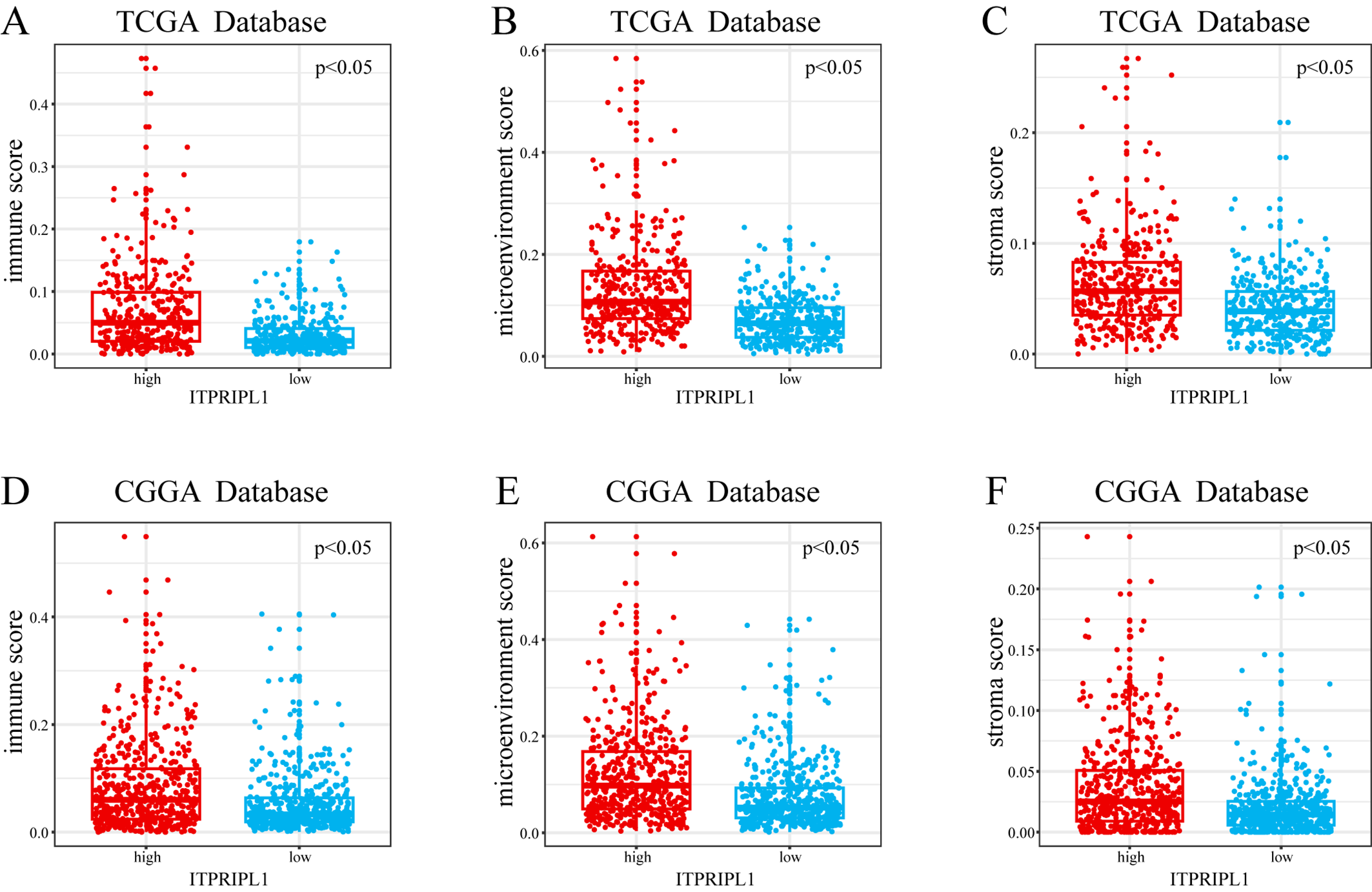
***

**Supplementary Figure 2. Impact of ITPRIPL1 expression on immune infiltration in glioma.** (A-C) Correlation between ITPRIPL1 expression and immune score, microenvironment score, and stromal score calculated using the xCell algorithm in the TCGA cohort. (D-F) Correlation between ITPRIPL1 expression and immune score, microenvironment score, and stromal score based on the xCell algorithm in the CGGA cohort.

***
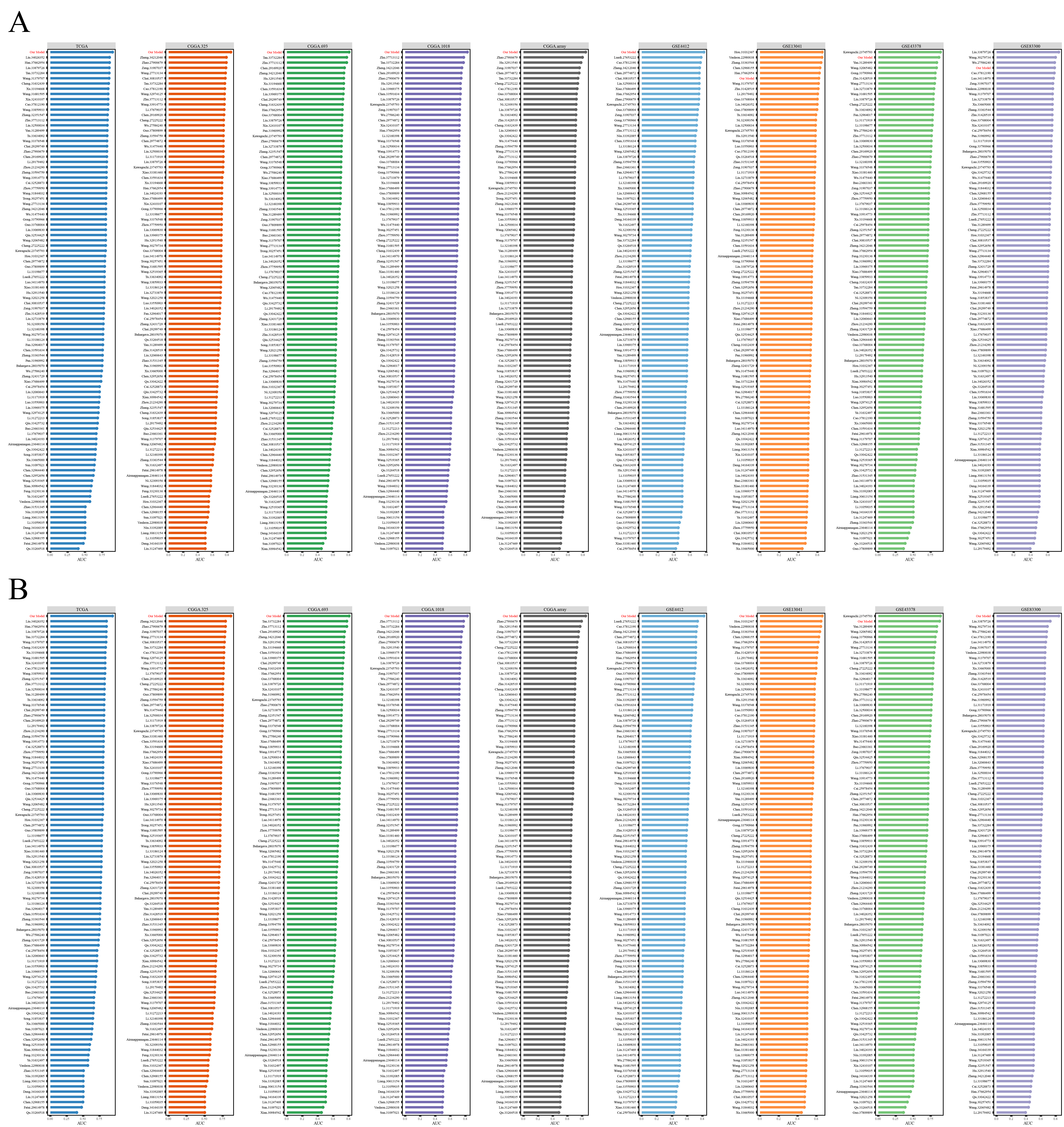
***

**Supplementary Figure 3. Comparison between the SuperPC model and 95 previously published glioma models.** (A) Comparison of 2-year AUC values between the SuperPC model and 95 published models. (B) Comparison of 3-year AUC values between the SuperPC model and 95 published models.
