# Supplementary material for: Immune Evasion Mechanism Mediated by ITPRIPL1 and Its Prognostic Implications in Glioma
Source: Brain Behav. 2025 Aug 12;15(8):e70762. doi: 10.1002/brb3.70762 (PMC12340539; doi:10.1002/brb3.70762)
Supplement: Supplementary file 2 — Supporting fig.4: brb370762‐sup‐0002‐SuppMat.docx [file BRB3-15-e70762-s003.docx]

***Supporting Information***

## Supplementary Figures

***
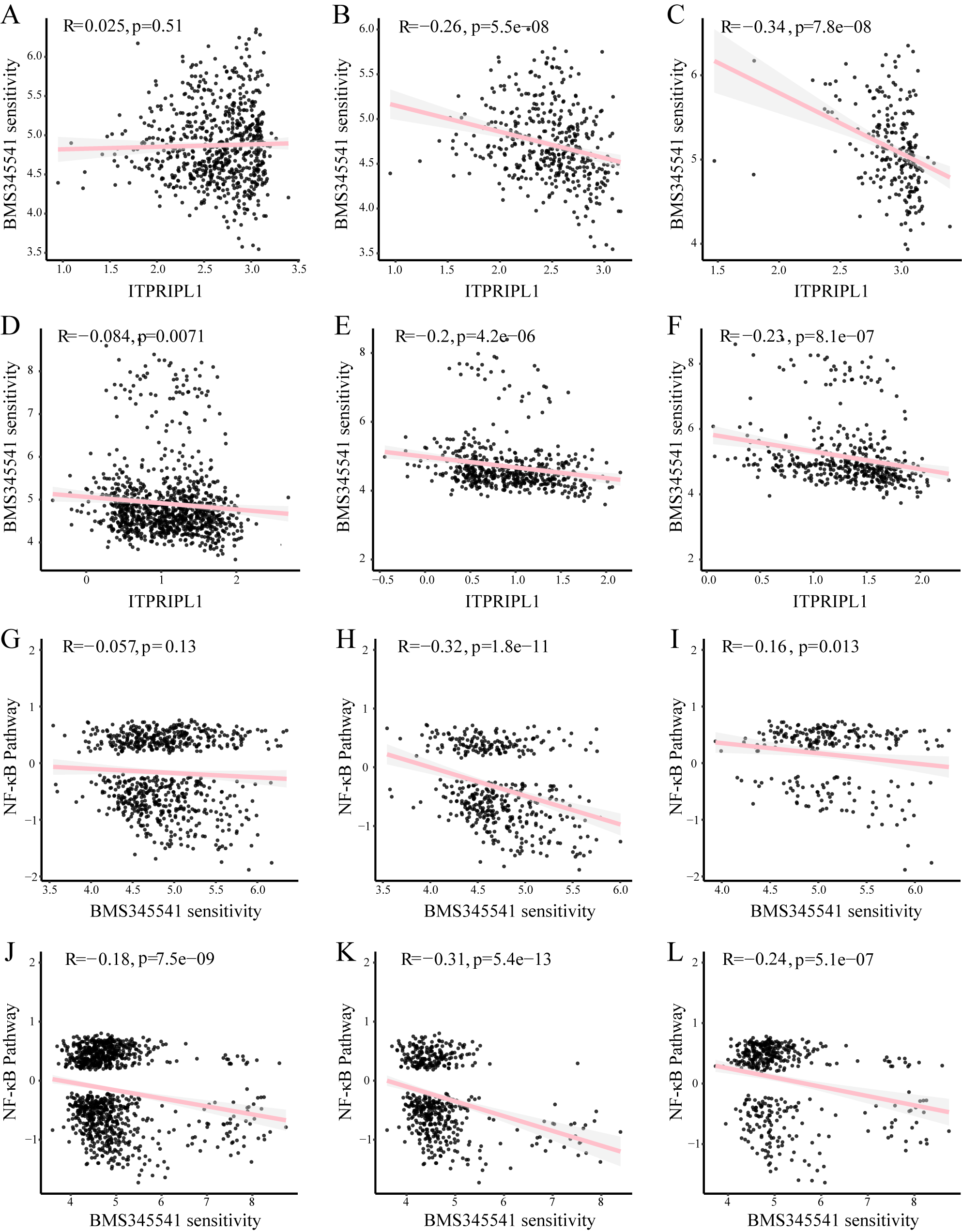
***

**Supplementary Figure 4. The effect of IDH status on BMS-345541-mediated inhibition of the NF-κB pathway.** (A-C) Correlation between BMS-345541 sensitivity and ITPRIPL1 expression in all patients, IDH-mutant patients, and IDH-wildtype patients in the TCGA cohort. (D-F) Correlation between BMS-345541 sensitivity and ITPRIPL1 expression in all patients, IDH-mutant patients, and IDH-wildtype patients in the CGGA cohort. (G-I) Correlation between BMS-345541 sensitivity and NF-κB pathway in all patients, IDH-mutant patients, and IDH-wildtype patients in the TCGA cohort. (J-L) Correlation between BMS-345541 sensitivity and NF-κB pathway in all patients, IDH-mutant patients, and IDH-wildtype patients in the CGGA cohort.
